# Bicuspidization of Insufficient Neoaortic Valve After Arterial Switch With Looping Coronary Anatomy

**DOI:** 10.1016/j.atssr.2023.03.005

**Published:** 2023-03-25

**Authors:** Megan L. Schultz, Glen S. Van Arsdell, Ming-Sing Si

**Affiliations:** 1 Division of Cardiac Surgery, Department of Surgery, University of California Los Angeles, Los Angeles, California

## Abstract

Neoaortic root dilation is inevitable in the years after an arterial switch operation, not uncommonly leading to neoaortic valve insufficiency that requires reoperation. Here we present a bicuspidization repair technique for addressing such neoaortic valve insufficiency in a child with the 1L 2RCx coronary pattern who underwent the arterial switch operation as a neonate for dextro-transposition of the great arteries that has good short-term outcomes thus far.

The arterial switch operation (ASO) is the standard treatment of dextro-transposition of the great arteries.^1^ Neoaortic root dilation commonly occurs in the years after ASO, occasionally leading to neoaortic insufficiency necessitating reoperation.^2^^,^^3^ A variety of procedures have been used to treat this type of neoaortic insufficiency, although current techniques often require nonnative tissue, prosthetic implants, or highly specialized surgical experience.^4^ Here we describe a neoaortic valve bicuspidization repair technique employed successfully in a patient with unusual coronary anatomy.

The patient, a 5-year-old boy with dextro-transposition of the great arteries, initially underwent a neonatal ASO with ventricular septal defect (VSD) repair at another institution. This was complicated by an injury to the right anterior cusp of the neoaortic valve. Re-repair of a residual VSD and replacement of the injured cusp with an allograft leaflet was performed 1 week later. The patient presented to our center with palpitations and chest discomfort. Echocardiography and cardiac magnetic resonance imaging demonstrated decreased excursion of the right anterior cusp, moderate neoaortic insufficiency, and dilated left ventricle. He was taken to the operating room for neoaortic valve repair vs replacement.

In the operating room, a dilated neoaortic root was encountered. The neoaortic valve had 2 thin and large native leaflets with a small, retracted right anterior (replacement) leaflet. Based on this anatomy, it was thought that the neoaortic valve could best be repaired with a bicuspidization technique. The patient had the 1L 2RCx coronary pattern, with the circumflex coronary artery arising off the right coronary artery (RCA) and looping posterior to the neoaortic root and crossing the sinus of the replacement leaflet. It was necessary to harvest an RCA button and thoroughly mobilize the circumflex coronary artery off the neoaortic root before performing the neoaortic valve bicuspidization (Figure A).

**Figure:**
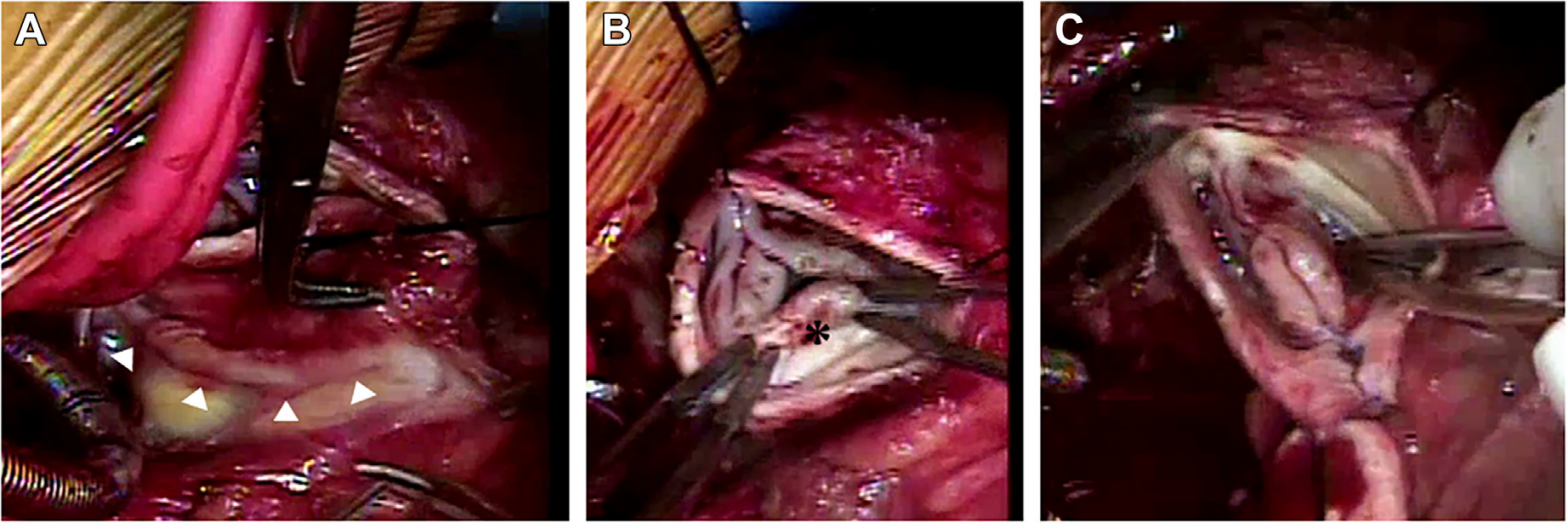
Intraoperative images. (A) Circumflex coronary artery dissected thoroughly off neoaortic root. Course denoted with arrowheads. (B) Appearance of the neoaortic valve before repair. Retracted right anterior cusp denoted with an asterisk. (C) Appearance of the bicuspidized neoaortic valve after repair.

Once the circumflex coronary artery was mobilized, attention was turned to the bicuspidization of the neoaortic valve. This was accomplished by excising the replacement right anterior leaflet and its sinus. A new commissural post was created in the area of the excised sinus, and the neoaortic root was reconstructed (Figures B, C). The previously harvested RCA button was reimplanted in a new location on the neoaortic root to prevent kinking of the vessel (Video).

Postoperative echocardiography revealed trace neoaortic insufficiency without stenosis, generous coaptation height, good biventricular function, and good flow in the RCA. The patient’s recovery was uncomplicated, and he was discharged home on postoperative day 3. The most recent follow-up echocardiogram available, 10 months postoperatively, has demonstrated trivial neoaortic valve insufficiency with 2 small regurgitant jets located at either end of the commissure. There is no neoaortic stenosis and excellent biventricular function, and the left ventricle has normalized in size.

## Comment

The ASO has become the standard treatment of transposition of the great arteries during the past several decades. It is performed routinely at experienced centers with low rates of operative and long-term mortality.^1^ However, the neoaortic root inevitably dilates out of proportion to somatic growth over time.^2^ Although this dilation does not always result in significant neoaortic valve regurgitation, the risk increases with time and with certain patient factors, such as presence of VSD at the time of ASO.^3^ A variety of surgical options can be employed for those patients who do require reintervention to address neoaortic insufficiency. Neoaortic valve replacement or Bentall is often performed,^4^ and consideration can be given to other techniques, such as repair with an annuloplasty ring or reverse Ross. The best procedure will depend on patient-specific disease, but valve replacement is rarely ideal, and a technique that fully allows for growth is best, for young patients especially.

We demonstrate an innovative technique for repairing the insufficient neoaortic valve in a patient with a history of ASO and unusual coronary anatomy. Neoaortic valve bicuspidization was well suited to this patient’s disease. Injury to the right anterior cusp at the time of his ASO required leaflet replacement, for which allograft tissue with no growth potential was used. Over time, the 2 native leaflets did grow, compensating for the lack of growth of the replacement leaflet. These 2 large native leaflets and the dilated neoaortic root provided the opportunity to excise the replacement leaflet along with its sinus without causing stenosis. In repairing this 5-year-old patient’s neoaortic valve with native tissue alone, growth potential was maintained.

Furthermore, it was critical to consider coronary anatomy in this patient. Approximately 30% of patients with transposition of the great arteries will also have an atypical coronary artery pattern,^5^^,^^6^ many of them with looping coronary anatomy as seen in our case. A thorough dissection of the proximal circumflex coronary and reimplantation of the RCA button in an appropriate location allowed bicuspidization of the valve and downsizing of the neoaortic root in this case while maintaining coronary patency.

In summary, we present here a bicuspidization technique that may be considered in patients with 2 good neoaortic leaflets and a dilated neoaortic valve annulus. Consideration of the coronary anatomy is paramount to the success of the repair technique. Although short-term results are favorable, continued follow-up of this repair technique is needed to assess its long-term durability.
